# Small cell carcinoma with neuroendocrine differentiation of subglottic larynx- a case report

**DOI:** 10.3389/fonc.2023.1222418

**Published:** 2023-09-25

**Authors:** Rafał Becht, Kajetan Kiełbowski, Justyna Żychowska, Wiktoria Dembowska, Małgorzata Król, Bożena Birkenfeld, Mateusz Owsiak, Magdalena Lewandowska, Jadwiga Kubrak, Katarzyna Amernik

**Affiliations:** ^1^ Department of Clinical Oncology, Chemotherapy and Cancer Immunotherapy, Pomeranian Medical University, Szczecin, Poland; ^2^ Department of Nuclear Medicine, Pomeranian Medical University, Szczecin, Poland; ^3^ Department of Diagnostic Imaging and Interventional Radiology, Pomeranian Medical University, Szczecin, Poland; ^4^ Department of Pathology, Pomeranian Medical University, Szczecin, Poland; ^5^ Department of Clinical Radiotherapy, West Pomeranian Oncology Center, Szczecin, Poland; ^6^ Department of Adult and Children Otolaryngology and Otolaryngological Oncology, Pomeranian Medical University, Szczecin, Poland

**Keywords:** small cell cancer, neuroendocrine neoplasm, laryngeal small cell cancer, laryngeal neuroendocrine cancer, laryngeal cancer

## Abstract

Small cell cancer (SCC) is a neuroendocrine neoplasm, which is most frequently found in the lungs. Extrapulmonary location of SCC is rare and may involve 2.5-5% of SCCs. We present a case of a 31-year-old male patient with an extremely uncommon subglottic SCC. The patient was qualified for a radical sequential chemoradiotherapy. After treatment, patient’s condition suggested complete remission. Recurrence was detected one year later, and the disease rapidly progressed, despite a second line chemotherapy. The patient died 29 months after initial diagnosis. This case aims to raise awareness on the aggressive laryngeal SCC and its good response to first line chemotherapy composed of cisplatin and etoposide, followed by radiotherapy.

## 1 Introduction

Small cell cancer (SCC) is an aggressive neoplasm, which is most frequently diagnosed in the lung (1). Extrapulmonary SCC (EPSCC) is rare, representing approximately 2.5% to 5% of SCCs (2). Genitourinary and gastrointestinal sites are the most common locations for EPSCC, while only 11% of these neoplasms develop in the head and neck region (3, 4). Simultaneously, SCCs represent less than 0.5% of head and neck cancers (5). Larynx is the most common location for SCC in the head and neck region, while supraglottis seems to be the most frequently involved laryngeal site (6). In this paper, we report an extremely rare case of SCC located in the subglottic larynx.

## 2 Case presentation

**Figure 1:**
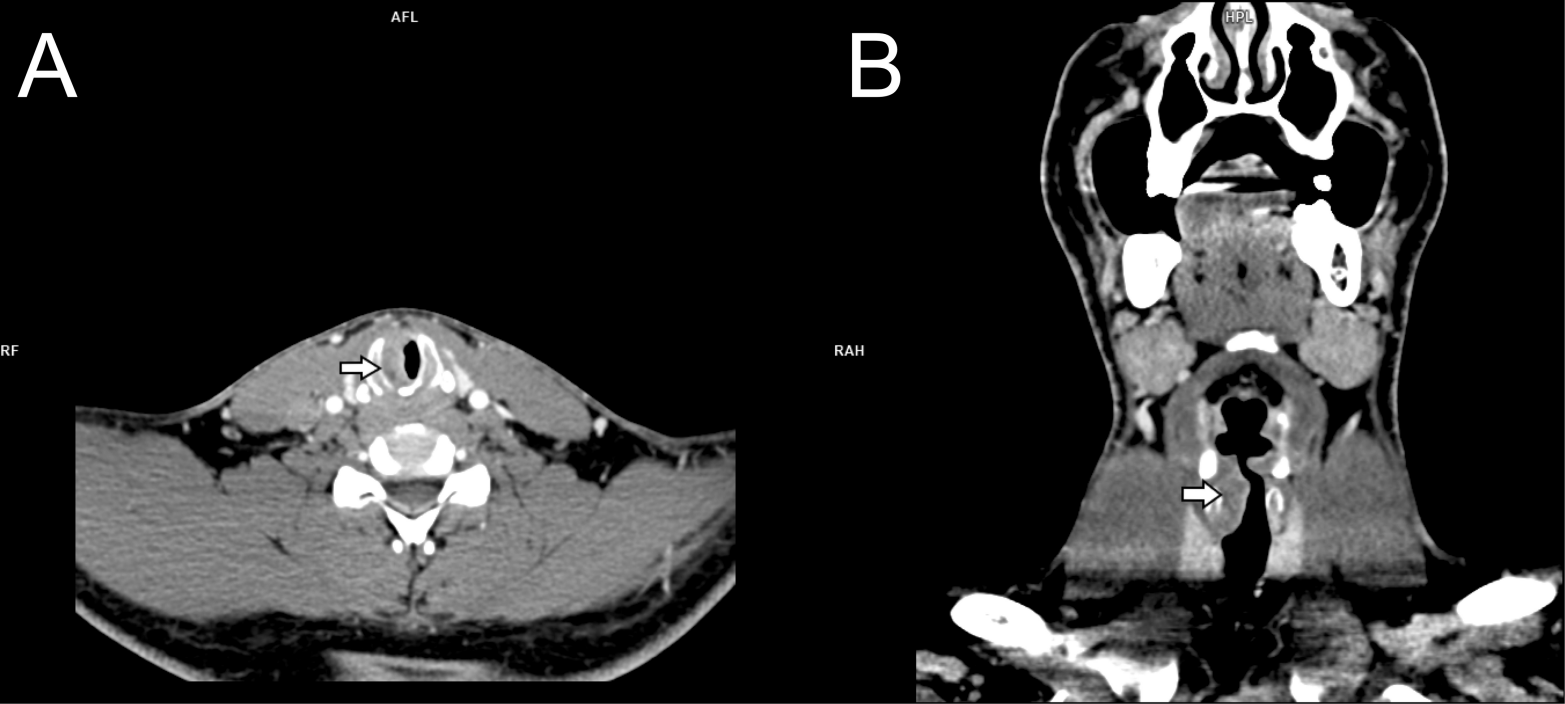
**(A)** Axial contrast-enhanced computed tomography shows right subglottic soft tissue mass (arrow) invading airway; **(B)** Coronal reformatted contrast-enhanced computed tomography shows mass (arrow) extension from right true vocal cord of glottis to the subglottic region.

**Figure 2:**
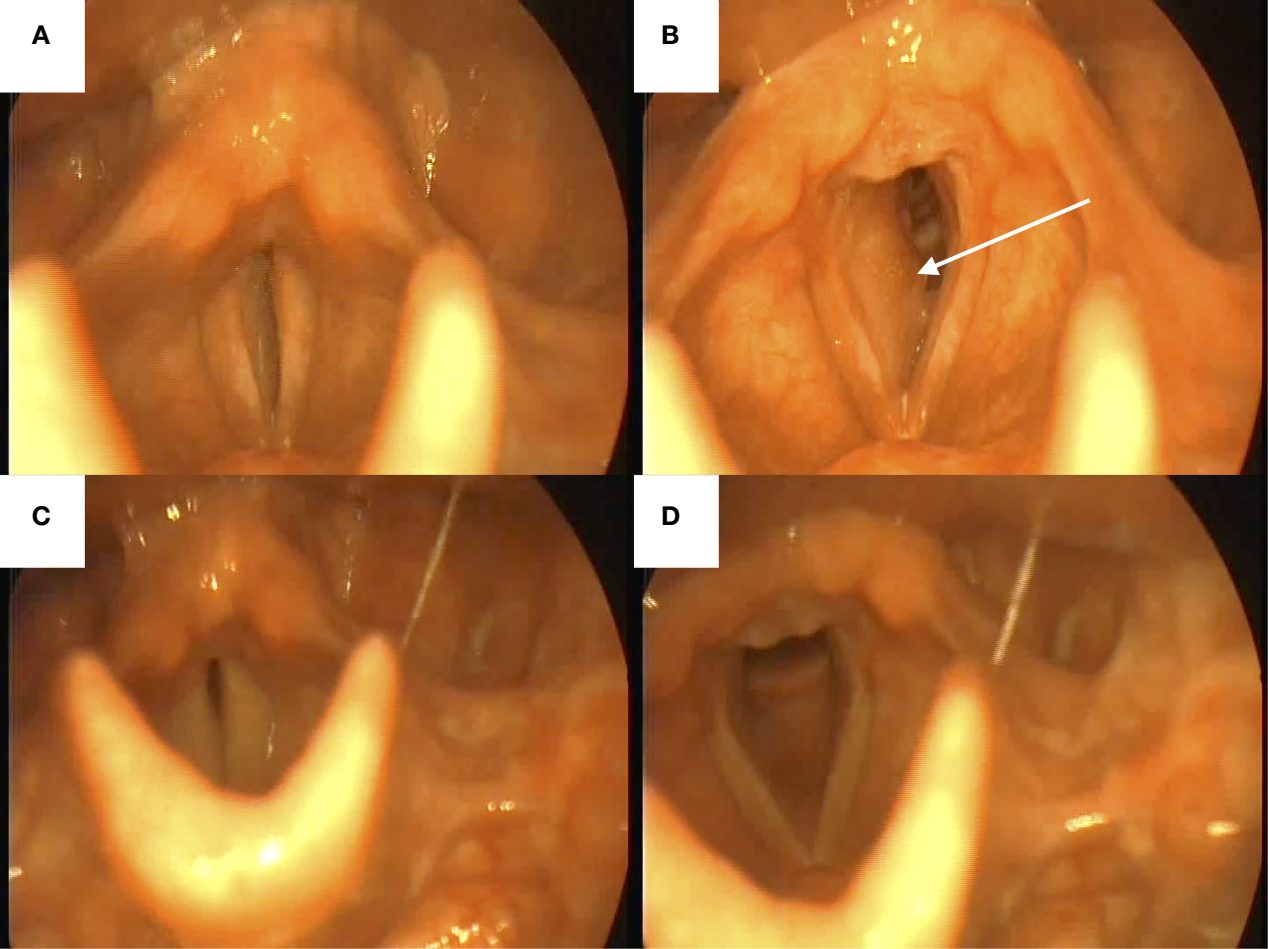
Views from nasofiberoscopy. **(A, B)** before the treatment with visible right-sided tumor (white arrow); **(C, D)** after the treatment.

In August 2016, a 31-year-old male patient with a long-history of cigarette smoking, alcoholism, and addiction to psychostimulants, experienced dyspnea, a sensation of foreign body in the throat and a deterioration of exercise tolerance. After consultation with a primary care physician, a chest x-ray was performed, but it showed no abnormalities. Due to the progressing symptoms and cervical lymphadenopathy, the patient was referred to the Department of Laryngology of the Pomeranian Medical University in Szczecin for diagnostic purposes. Physical examination revealed subglottic laryngeal stenosis and laryngeal dyspnea. A computed tomography (CT) revealed a heterogenous lesion in the subglottic and glottal parts on the right side, measuring 13 x 18 x 25 mm (**Figure 1**). The lesion demonstrated contrast-enhancement. The lesion extended beyond the midline, narrowing the lumen of the larynx. It covered the subglottic region, right vocal fold, anterior and posterior commissures, shallowing the right laryngeal ventricle (**Figure 2**). The lesion was adjacent to the cricoid, thyroid, and arytenoid cartilages, without features of infiltration. Furthermore, bilateral enlargement of the IIA lymph nodes was observed (right side 13 x 9 mm, left side 13 x 7 mm). The lesions did not have any obvious features of metastasis. Right-sided lymph nodes from groups III and IV were observed (the largest measured 13 x 10 mm and surrounded jugular vein). CT of the chest, abdomen and pelvis did not show any abnormalities.

**Figure 3:**
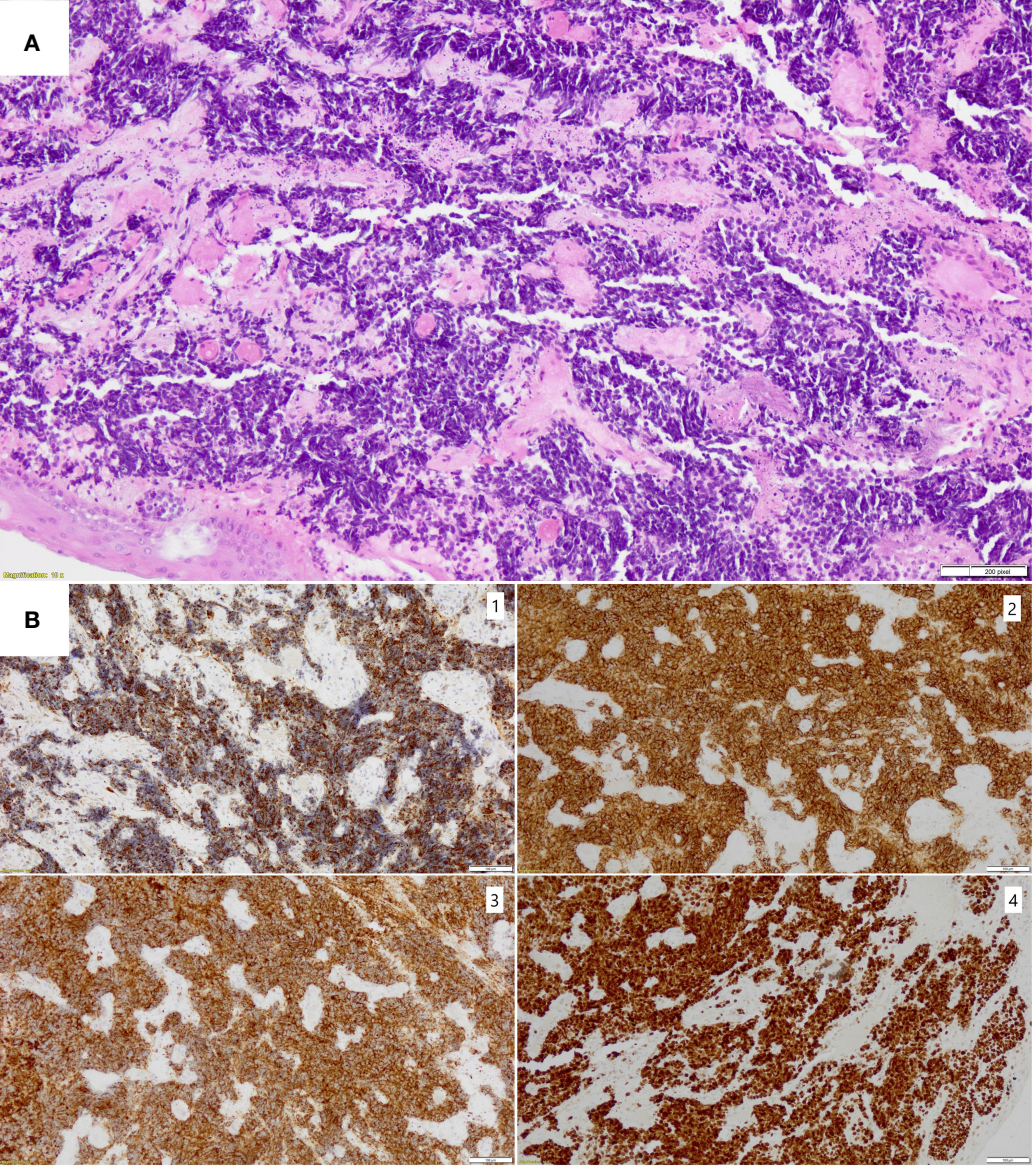
**(A)** Hematoxilin and eosin staining of small cell carcinoma with significant crushing artifact (magnification x10); **(B)** Immunohistochemical stainings confirming diagnosis of small cell carcinoma with neuroendocrine differentiation (magnification x10): 1. CKAE1/AE3 with dot-like pattern, 2. CD56- strong positive membranous staining, 3. synaptophysin- strong positive cytoplasmic staining, 4. Ki67- positive nuclear staining in 99% of tumor cells.

Subsequently, a biopsy of the subglottic area was performed. Histopathologic examination revealed infiltration of small cell neoplasm with the immunophenotype CKAE1/AE3+ “dot like”. Neoplastic cells showed positive synaptophysin, CD56, and non-specific TTF1 immunohistochemical expression, while immunostainings for chromogranin and LCA were negative (**Figure 3**). Proliferative index Ki67 was 99%.

**Figure 4:**
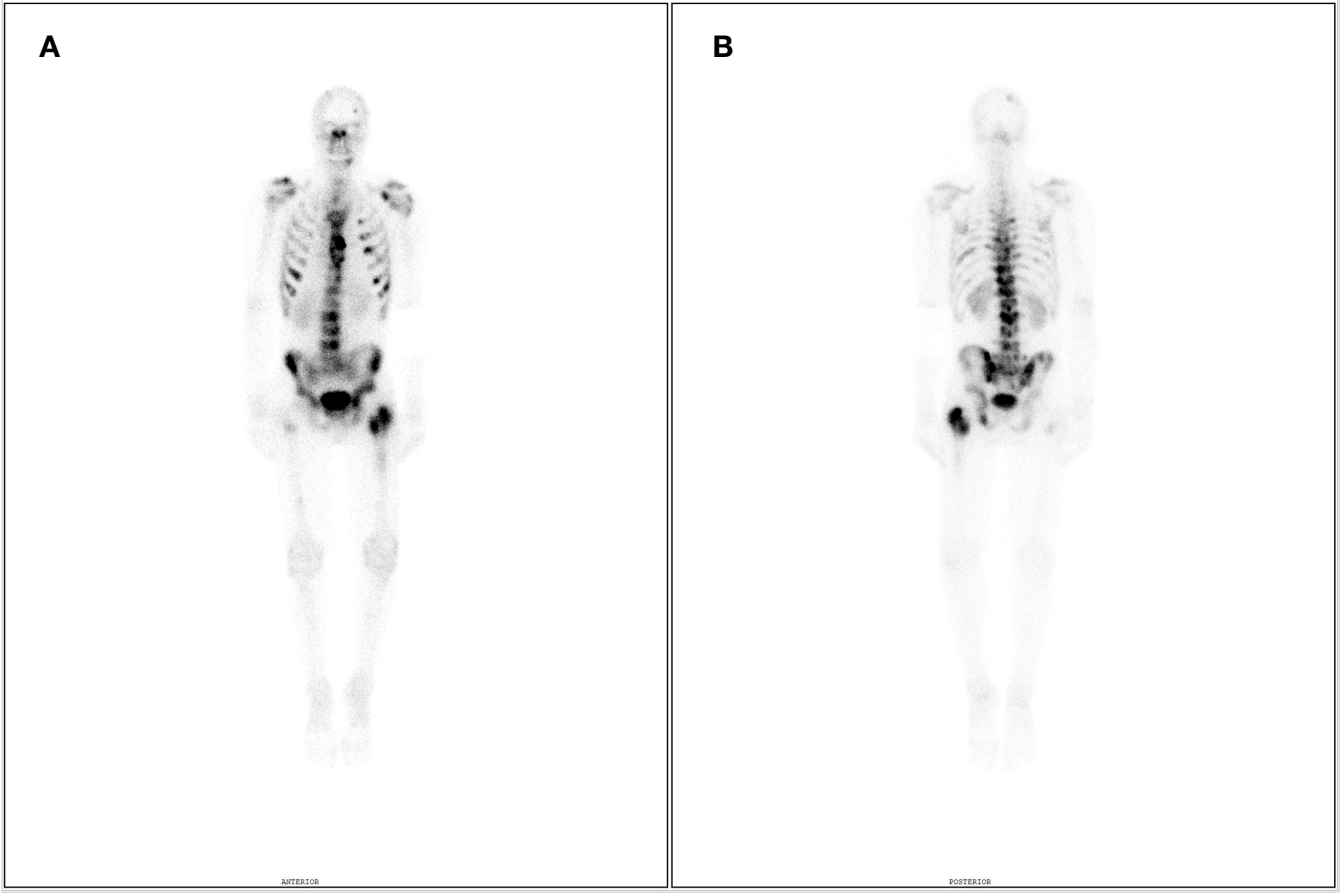
Skeletal scintigraphy in **(A)** A-P and **(B)** P-A views after Tc99m MDP administration. Numerous metastatic foci within the spine, pelvis, ribs, skull, proximal end of the right femur, and where the prosthesis enters the left femur. Very fast progression of the disease.

As a result, a small cell neuroendocrine cancer was diagnosed. To establish a clinical stage of the disease, a positron emission tomography (PET-CT) was performed, which confirmed enhanced metabolic activity of the lesion in the laryngeal part of the pharynx and lymph nodes from groups III and IV, without metastasis. Subsequently, the patient was presented to a multidisciplinary team, which discussed the treatment strategy. Firstly, due to a significant dyspnea, tracheostomy was performed. Nevertheless, the patient was in a good clinical condition with ECOG-0 performance status. Furthermore, failure of other organs or systems was not detected. The patient was qualified for a radical sequential treatment composed of PE regimen (cisplatin 30 mg/m^2^, etoposide 100 mg/m^2^) every 21 days, followed by radiotherapy in a sequential model. Throughout 5 months of treatment, the patient received 6 cycles of PE chemotherapy in respective doses with acceptable tolerance. Subsequently, in June 2017, the patient received radical radiotherapy (2.2 Gy/dose, 66 Gy in 30 doses) with good tolerance. In November 2017, a control CT scan of the neck and chest was normal, there were no signs of recurrence. Therefore, patient’s condition suggested complete remission (CR). Unfortunately, due to the temporary change of residence, the patient has been lost to follow-up. In September 2018, a follow-up laryngeal CT revealed a contrast enhancement of the right vocal cord and subglottic mucosa (2 mm thickness), which suggested a relapse. Subsequently, a chest x-ray revealed a circular shadow measuring 29 mm. It was followed by a chest CT, which confirmed disseminated metastasis-like lesions in mediastinum and lungs. Thirty-one mm lesion in the upper part of the right hilum, a 3 mm nodule in the right sixth pulmonary segment and a 2 mm nodule in the right ninth segment were observed. Histopathological examination of sample from the right bronchial mucosa confirmed CD56 + small cell cancer infiltration with Ki67 measuring 80%. The patient also reported pain of the left hip and bone scintigraphy was performed (800 MBq MDP labeled with Tc99m). Elevated uptake in the left femoral head, neck, and in the region of the trochanters were observed. Pelvis CT revealed a pathologic fracture of the left neck of the femur. Consequently, the patient underwent a total left hip alloplastic joint replacement. Intraoperative samples were collected for a histopathological examination, which confirmed metastasis of small cell neuroendocrine cancer. Subsequently, In January 2019, the patient was given a palliative intensity-modulated radiation therapy (20 Gy, 5 fractions) in the region of iliac nerve. In February and March, metastatic lesions were confirmed in the right lung, liver, and bones. Since February, the patient has been given a palliative chemotherapy in the CAV protocol (cyclophosphamide 800 mg/m^2^, doxorubicin 50mg/m^2^, and vincristine 1.4 mg/m^2^). In February, a periprosthetic tissue destruction was observed and a single-photon emission computerized tomography was performed (SPECT, 780 MBq MDP labeled with Tc99m). It confirmed multiple lesions with elevated uptake in the axial skeleton, ribs, pelvis, and structures of the left hip joint (**Figure 4**). A chest x-ray from March 2019 showed a tumor measuring 48 mm in the right hilum and a shadow in the II right rib measuring 19 mm. Due to progressing pain and dyspnea, the patient was unable to lie down. Abdomen ultrasonography revealed multiple hyperechogenic focal lesions up to 14 mm in diameter. The patient received only 3 cycles of CAV chemotherapy due to a progression of the disease and a worsening of the clinical condition (ECOG-3). As a result, chemotherapy was discontinued, and the patient was transferred to a palliative care. The patient died in May 2019 (**Table 1**).

**Table 1:** Medical history of presented patient.

Time	Relevant clinical information
August 2016	Beginning of symptoms
January 2017	Diagnosis of laryngeal small cell cancer
January 2017	Qualification for the sequential therapy (PE chemotherapy + radiotherapy)
November 2017	Complete response after treatment
September 2018	Recurrence
January-March 2019	Palliative radio- and chemotherapy (CAV protocol)
–	Disease progression and worsening of the clinical condition throughout treatment
May 2019	Death

## 3 Discussion

According to the WHO, laryngeal neuroendocrine cancers (NEC) are classified into well-differentiated, moderate, and poorly differentiated cancers. The latter is further divided into small cell NEC and large cell NEC (7). Small cell cancers represent only 0.3% of all head and neck cancers (5). They form a heterogenous group of neoplasms, which is challenging to diagnose and successfully treat. Neuroendocrine tumors are thought to originate from enterochromaffin (Kulchitsky) cells (6, 8). The most common location of SCC is the lung, while smoking is the most significant risk factor (9). In contrast, it seems that cigarettes are less significant in the extrapulmonary variant of the disease (9, 10). The disease may manifest with multiple symptoms, including hoarseness, dyspnea, or dysphagia (11). Poorly differentiated NECs are associated with elevated mitotic activity and necrosis (12). Differential diagnosis for laryngeal SCC is broad and may include large cell NEC, squamous cell carcinoma, paraganglioma, extended thyroid medullary carcinoma, or sarcomas (13), among others. As a result, immunohistochemistry helps in establishing a proper diagnosis. In the presented case, neoplastic cells showed positive expression of synaptophysin, CD56, and non-specific thyroid transcription factor 1 (TTF1), while they were negative for chromogranin and LCA. CD56, also known as neural cell adhesion molecule (NCAM) is expressed by various cells throughout the human body, including neural and immune cells. Importantly, they are also expressed in hematological malignancies (multiple myeloma) and solid tumors (14, 15). CD56, together with chromogranin and synaptophysin represent typical markers for a neuroendocrine tumor (13). SCCs might also express TTF-1 (16, 17). Moreover, Salhab et al. demonstrated that 12/34 cases of extrapulmonary SCC express programmed death ligand 1 (PD-L1), while other markers are being proposed for the diagnosis of SCC in various locations (18, 19).

Treatment of SCC of head and neck origin may include the combination of chemotherapy, surgery, and radiotherapy (5). The combination of treatment methods is considered more effective and due to the rarity of the disease, strategy is similar to the treatment of small cell lung cancer (20). According to the ESMO guidelines, the first line chemotherapy in small cell lung cancer is composed of cisplatin/carboplatin with etoposide. SCC is sensitive to chemotherapy, but the relapse frequently occurs within six months. A second line treatment may include topotecan, CAV (doxorubicin, cyclophosphamide, vincristine), or lurbinectedin, among others. Moreover, immunotherapy has been shown to play an enormous role in SCC treatment and several agents are recommended in the treatment strategy as well (21).

In our case, the first line chemotherapy was PE regiment (cisplatin, etoposide). According to van der Laan and colleagues, the preferred primary treatment for laryngeal SCC is chemotherapy and radiotherapy, and this strategy was the most common in their analysis, followed by surgery and radiotherapy with surgery (34.4%, 15.9%, and 14%, respectively) (22). Chemotherapy is usually composed of cisplatin or carboplatin with etoposide (23–25). Zhou et al. reported a patient with nasopharyngeal small cell neuroendocrine tumor (T4N2M0, IVA), who was treated with radiotherapy (a total of 66Gy, 33 fractions) and chemotherapy (cisplatin, etoposide) and achieved a complete remission for 46 months (26). Raposo et al. published a report of a patient with laryngeal SCC treated with radiotherapy and chemotherapy (cisplatin, etoposide), who experienced recurrence and required additional cycles of treatment, together with laryngectomy, and survived for 47 months (27). Iqbal and colleagues published a case series of 9 patients with laryngeal small cell neuroendocrine cancers, among whom one patient survived for 99 months (28). Nevertheless, these few extraordinary cases seem to be rarity, as prognosis in laryngeal SCC is poor. Five-year survival rate ranges from 5% to approximately 20% (22, 28–30). In the presented case, the patient started experiencing symptoms in August 2016, while remission was recorded in November 2017. Recurrence was detected in September 2018 and death occurred in May 2019, which indicated that the patient survived 29 months from the diagnosis.

Classic chemotherapeutics used in the treatment of SCC are long-known anticancer agents. Therefore, there is a need for new treatment agents. Since SCC cells might express PD-L1, these cancers may be treated with immunotherapy. Immune Checkpoint Inhibitors (ICIs) are monoclonal antibodies, which target PD-1/PD-L1 pathway or cytotoxic T-lymphocyte associated protein 4 (CTLA-4), proteins that induce immunosuppression. Naqvi and colleagues reported a case of laryngeal SCC, who underwent treatment with carboplatin and etoposide. After recurrence, the patient was treated with nivolumab (anti-PD-1) and ipilimumab (anti-CTLA-4), which resulted in complete resolution of the disease (31). Atezolizumab is a novel monoclonal antibody targeting PD-L1. Recent phase 3 clinical trial evaluated its use with carboplatin and etoposide in the treatment of small cell lung cancer. The study concluded that the addition of atezolizumab in the first-line chemotherapy resulted in improved overall and progression-free survival (32). Niforatos et al. described the use of atezolizumab with cisplatin and etoposide in a patient with head and neck small cell neuroendocrine cancer with good response (33). Moreover, durvalumab (anti-PD-L1) has been showed to significantly improve overall survival in patients with an extensive stage small cell lung cancer in a recent phase 3 clinical trial (CASPIAN) (34). Importantly, pembrolizumab (anti-PD-1) significantly improved progression free survival in patients with an extensive stage small cell lung cancer (KEYNOTE-604) (35). It was not possible to introduce immunotherapy in the presented patient due to the lack of registration indications at that time.

## 4 Conclusions

Primary small cell neuroendocrine cancer of the subglottic larynx is a rare entity among head and neck cancers. The disease has an aggressive character, but also a good response to the first line chemotherapy, composed of cisplatin and etoposide, which are long-known and efficient agents in the treatment of SCC. Nevertheless, a relapse represents a significant challenge for successful treatment. Novel treatment agents are required for the management of refractory laryngeal SCCs. Additionally, to improve the treatment outcomes in patients with resistant or refractory disease, a precise and personalized diagnosis is required, which will give the possibility of targeted therapy.

## Data availability statement

The original contributions presented in the study are included in the article. Further inquiries can be directed to the corresponding author.

## Ethics statement

Ethical approval was not required for the studies involving humans because according to local regulations and after consultation with the Bioethics Committee, no approval is required for “case report” work. The patient gave written consent to the case description during treatment. The studies were conducted in accordance with the local legislation and institutional requirements. The participants provided their written informed consent to participate in this study. Written informed consent was obtained from the individual(s) for the publication of any potentially identifiable images or data included in this article.

## Author contributions

Concept of the study: RB. Data Collection: RB, ML, BB, MO, JK, and KA. Manuscript Drafting: RB, KK, JZ, MK, WD, BB, MO, ML, JK, and KA. Manuscript Revision: RB, KK, JZ, MK, WD, BB, MO, ML, JK, and KA. Supervision: RB. All authors contributed to the article and approved the submitted version.
